# Adult Living Donor Liver Transplantation with ABO-Incompatible Grafts: A German Single Center Experience

**DOI:** 10.1155/2009/759581

**Published:** 2010-02-03

**Authors:** Armin D. Goralczyk, Aiman Obed, Andreas Schnitzbauer, Axel Doenecke, Tung Yu Tsui, Marcus N. Scherer, Giuliano Ramadori, Thomas Lorf

**Affiliations:** ^1^Department of General and Visceral Surgery, University Medical Center Göttingen, 37099 Göttingen, Germany; ^2^Department of Surgery, Universitätsklinikum Regensburg, Franz-Josef-Strauß-Allee 11, 93053 Regensburg, Germany; ^3^Department of Hepatobiliary and Transplant Surgery, Universitätsklinikum Hamburg-Eppendorf, Martinistraße 52, 20246 Hamburg, Germany; ^4^Department of Gastroenterology, University Medical Center Göttingen, 37099 Göttingen, Germany

## Abstract

Adult living donor liver transplantations (ALDLTs) across the ABO blood group barrier have been reported in Asia, North Americas, and Europe, but not yet in Germany. Several strategies have been established to overcome the detrimental effects that are attached with such a disparity between donor and host, but no gold standard has yet emerged. Here, we present the first experiences with three ABO-incompatible adult living donor liver transplantations in Germany applying different immunosuppressive strategies. Four patient-donor couples were considered for ABO-incompatible ALDLT. In these patients, resident ABO blood group antibodies (isoagglutinins) were depleted by plasmapheresis or immunoadsorption and replenishment was inhibited by splenectomy and/or B-cell-targeted immunosuppression. Despite different treatments ALDLT could safely be performed in three patients and all patients had good initial graft function without signs for antibody-mediated rejection (AMR). Two patients had long-term graft survival with stable graft function. We thus propose the feasibility of ABO-incompatible ALDLT with these protocols and advocate further expansion of ABO incompatible ALDLT in multicenter trials to improve efficacy and safety.

## 1. Introduction

Adult-to-adult living donor liver transplantation (ALDLT) has been established as an excellent treatment method for patients with end-stage liver disease and has achieved exponential growth, especially in Asia [1–4]. Graft and patient survival now are currently equal to those from cadaveric transplantation [4]. 

However, some patients who have otherwise suitable living donors are not transplanted because of ABO blood group incompatibility. In the 1960s, it was shown that transplantation of solid organs, especially kidneys, across the blood group barrier is associated with hyperacute rejection and reduced graft survival [5–8]. Therefore, in most countries, ABO incompatible liver transplantation was generally only accepted for cases of immediate need and unavailability of a compatible graft. Interestingly, early reports of blood group incompatible liver transplantation have shown that a surprisingly large number of grafts were successful, and it has thus been suggested that the liver may be less prone to hyperacute rejection than other solid organs [9, 10]. 

Since graft survival was still unacceptably low, new therapeutic strategies to ameliorate ABO-incompatible ALDLT were necessary. In the 1990s, these methods were investigated, mainly in Asia due to the shortage of deceased donors in these countries and the frequent blood group mismatch between living donor and recipient [11]. With the new strategies, survival has improved considerably and has reached 70% at three years in the latest cohort [12]. Recently, some authors even report similar outcomes of ABO-incompatible ALDLT as compared to compatible ALDLT [13, 14]. ABO-incompatible ALDLT has subsequently gained acceptance in Europe, namely, in Sweden [15, 16] and Belgium [17]. 

Since the recipient is (by definition) presensitized, the recognition of blood group antigens by preformed recipient isoagglutinins and its detrimental consequences remain the central problem of ABO-incompatible transplantation [7, 18]. Unlike in kidney and heart transplantation, hyperacute rejection, especially against MHC-class-I antigens, is rarely observed clinically in liver transplantation [19]. Antibody-mediated rejection (AMR) may still be present, but it usually manifests within 2 to 4 weeks after transplantation [19]. In AMR, the preformed isoagglutinins bind to the graft vasculature, resulting in complement activation, migration of neutrophils, vessel damage, diffuse intravascular thrombosis, and consequent activation of the fibrinolytic system with hemorrhagic necrosis of the graft [19]. In addition to AMR, a high incidence of hepatic artery and biliary complications can be found in patients receiving an ABO-incompatible allograft [11, 20]. It has been suggested that this also may be due to immunologic injury, since blood group antigens can also be found on bile duct epithelium [20]. 

In most clinical studies, two main strategies to reduce antibody-mediated complications have been tested in combination. First, preformed isoagglutinins in the recipient are reduced before transplantation by apheresis [11, 21, 22] or immunoadsorption [17, 23, 24]. Second, restoration of isoagglutinins by plasma cells is suppressed by splenectomy, reinforced immunosuppression [11], or by specifically interfering with the maturation [17, 21, 25–28] or activation [29, 30] of B cells. 

Here, we report our experiences with ABO incompatible ALDLT from a single center in Germany applying different aforementioned strategies. We wish to present our practical experience, including heterogenic results to the transplant community to show that this strategy is feasible and merits further expansion.

## 2. Patients and Methods

### 2.1. Patients and Donors

This case series includes four patients, of which three underwent ALDLT between June 2001 and September 2007. Clinical characteristics of the patients and donor-recipient blood group match are shown in Table 1.

All four patients had either end-stage liver disease or malignant disease of the liver without the possibility of complete resection, while extrahepatic tumor spreading was excluded by intense staging procedures. Patients were evaluated for standard liver transplantation and placed on the waiting list. As a standard practice of our institution, the patients were extensively informed about the possibilities and risks of ALDLT. We were then contacted by the patients and a possible donor on their own behalf to initiate evaluation for ALDLT. Although blood group incompatibility was found early in the evaluation process, we continued evaluation after informed consent due to disease severity. In all four patient-couples, recipient and donor were found medically suitable, while the interdisciplinary ethics committee on living donor liver transplantation found no contraindications according to German transplantation law. Consecutively, ALDLT was performed.

### 2.2. Blood Group Typing and Antibody Titers

Red blood cell group was determined with commercially available antisera according to standard immunohematologic techniques. The Anti-A1, -A2, and -B antibodies were determined with direct and indirect isoagglutination assays. Test erythrocytes were suspended in serial doubling dilutions of recipient serum, centrifuged, and analyzed for agglutination. This assay was defined as representing immunoglobulin (Ig) M activity. Specimens were then incubated at 37°C for 30 minutes and washed with normal saline, and antihuman globulin (Coombs) antiserum was added. The specimen was then centrifuged and analyzed for agglutination. This antiglobulin assay was defined as representing IgG activity. By standard red cell agglutination readings, the most dilute unequivocally positive reaction was defined as the anti-A or -B antibody titer value in each assay.

### 2.3. Perioperative Plasmapheresis

In all patients, plasmapheresis was used to reduce isoagglutinin titers to 1 : 16 or lower before and after ALDLT. A standard apheresis system was used for antibody depletion. Isoagglutinin titers were measured before plasmapheresis, after plasmapheresis, and on the following day to assess the efficacy of plasmapheresis and the need for additional filtration. In patient 3 immunoadsorption (IA) was also used to reduce antibody titers. The same standard apheresis system, although not specifically designed for column treatments, could easily be adjusted for this purpose. Plasma was recirculated through blood group A/B carbohydrate antigen columns (Glycosorb, Glycorex AB, Lund, Sweden) and then retransfused to the patient. The method has been described in detail by Kumlien and colleagues [23].

### 2.4. Immunosuppression

**Figure 1:**
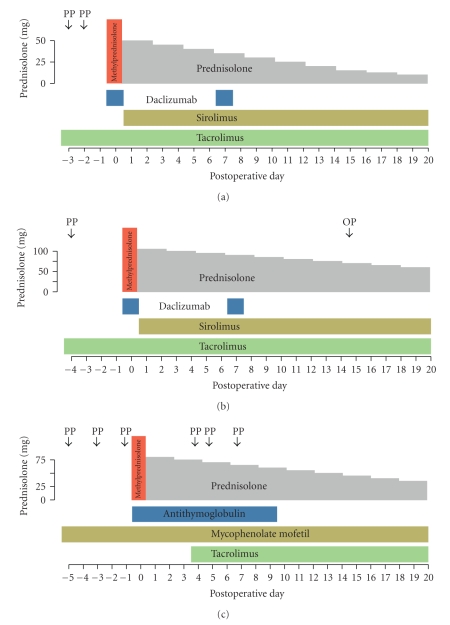
Immunosuppressive regimens of patients 1, 2, and 4 (panels (a)–(c), resp.) from the day of first preoperative treatment until postoperative day 20. Arrows indicate plasmapheresis or immunoadsorption; grey, green, and khaki colored areas correspond to maintenance therapy with prednisolone, tacrolimus, and adjuvant immunosuppression, respectively; blue and red area corresponds to induction treatment with daclizumab (100 mg at ALDLT and 50 mg on the seventh postoperative day) or antithymocyte globulin (1.5 mg per kilogram body weight) for 10 days and methylprednisolone (500 mg), respectively. PP: plasmapheresis; OP: relaparotomy.

The immunosuppressive regimens used are very heterogenic due to the long-time period over which these four ABO-incompatible ALDLTs were performed. An overview of the patients' immunosuppressive regimen is shown in Figure 1. The standard immunosuppressive regimen consisted of induction therapy with antibodies directed against white blood cell epitopes, maintenance therapy with corticosteroids and tacrolimus (Prograf, Astellas Pharma, Tokyo, Japan), and adjuvant immunosuppression with either sirolimus (Wyeth, Madison, USA) or mycophenolate mofetil (CellCept, Roche Pharmaceuticals, Basel, Switzerland). 

Therapy with steroids was initiated at surgery intraoperatively (500 mg methylprednisolone) and continued at 1 mg/kg body weight prednisolone tapered by 5 or 2.5 mg every two days until a maintenance dose of 7.5 mg was reached. In the first two patients, therapy with tacrolimus was started as a preemptive immunosuppression three days before ALDLT at half the standard dose (0.05 mg/kg body weight), continued after ALDLT at full dose (0.1 mg/kg body weight), and adjusted to achieve a trough plasma level of 8–10 *μ*g per liter. In the other two patients, standard immunosuppressive protocol at our institution had changed to a tacrolimus sparing regimen to ameliorate the detrimental effects of tacrolimus on renal function. Tacrolimus was administered on the fourth postoperative day, starting with a dose of 0.01 mg/kg body weight per day and increasing the daily dose by 1-2 mg according to renal function to achieve a trough plasma level of 8–10 *μ*g per liter. 

In the first two patients (Figures 1(a) and 1(b)), splenectomy was performed to reduce resident B cells in the recipient before implantation of the graft. At the time of transplantation and on the seventh postoperative day, the interleukin (IL)-2 receptor antagonist daclizumab (Zenapax, Roche Pharmaceuticals, Basel, Switzerland) was infused at 100 mg and 50 mg, respectively, for the induction of immunosuppression. As an additional immunosuppressant, sirolimus was administered at 5 mg per day. 

The third patient was treated with rituximab (MabThera, Roche Pharmaceuticals, Basel, Switzerland) at 375 mg per square meter body surface only once, after extrahepatic spread of malignant disease had been excluded by explorative laparotomy. Mycophenolate mofetil was administered for adjuvant immunosuppression at 1 g every 12 hours starting 5 days before planned ALDLT. Since the patient was eventually not transplanted (see below results), he received neither steroids nor tacrolimus. 

The fourth patient (Figure 1(c)) received 1.5 mg per kilogram body weight antithymocyte globulin (ATG, Thymoglobulin, Genzyme Corporation, Cambridge, USA) every 24 hours for 10 days starting on the day of transplantation. Mycophenolate mofetil was given at 1 g every 12 hours starting 5 days before transplantation. No splenectomy was performed in the last two patients (patients 3 and 4).

## 3. Results

### 3.1. Isoagglutinin Titers

**Figure 2:**
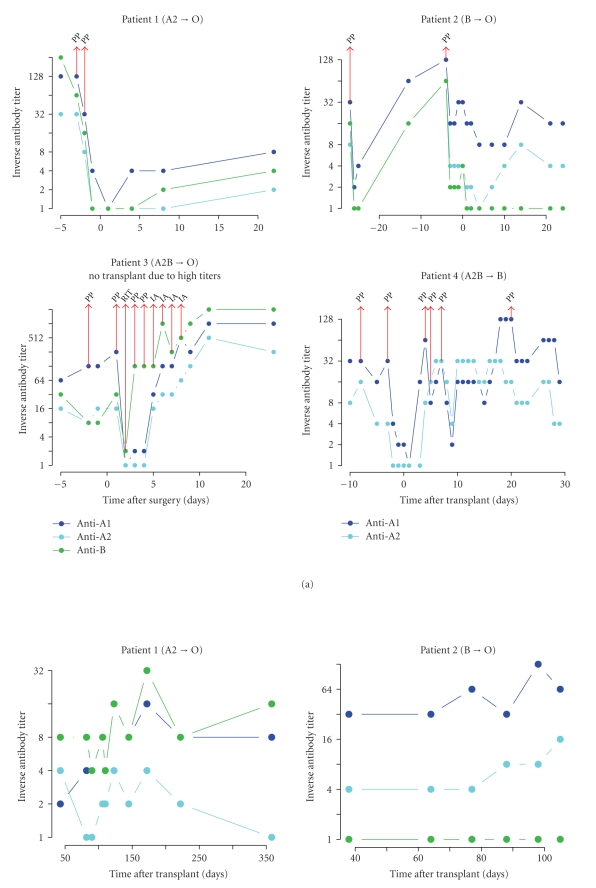

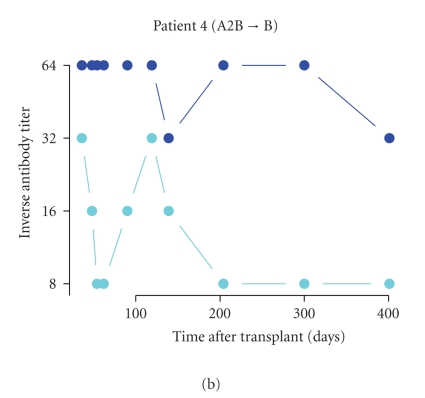
Perioperative isoagglutinin titers (early phase, panel (a); late phase, panel (b)). Inverse antibody titers of type Anti-A1, -A2, and B are shown in dark blue, light blue, and green, respectively, on a logarithmic scale. The time scale indicates days after transplant (patients 1, 2, and 4) or days post surgery (patient 3). IA: immunoadsorption; PP: plasmapheresis; RIT: rituximab).

In all patients except patient 3, isoagglutinin titers could be lowered below 1 : 16 by two apheresis sessions before ALDLT (see Figure 2(a)). In patient 3, apheresis and immunoadsorption had no long-term effect. Although isoagglutinins could effectively be depleted by plasmapheresis and immunoadsoption (comparing isoagglutinin titers measured before and directly after the session), an immediate rebound effect occurred overnight; because of this unusual course and double ABO-incompatibility, we decided against ALDLT after eight unsuccessful treatments. 

In the first 30 days after ALDLT, isoagglutinin titers remained below 1 : 16 without further treatment in the first two patients. Rising titer levels above the predefined threshold of 1 : 16 made repeated plasmapheresis necessary in patient 4. We decided against further treatment after the fourth session because he had stable graft function without signs of rejection. Long-term titer levels remained stable in all three transplanted patients without further treatment (see Figure 2(b)).

### 3.2. Surgery and Graft Function

Donor hepatectomy and transplantation was uneventful in all three donor-recipient couples. Serious adverse events or complications were not observed in any of the donors. Initial liver function tests indicated good graft function in all three transplanted patients.

### 3.3. Adverse Events

We observed one febrile nonhemolytic transfusion reaction at the first plasmapheresis session of patient 3 (patient finally not-transplanted). Plasmapheresis was stopped immediately and the symptoms resolved within 12 hours. No other adverse events associated with plasmapheresis occurred in any of the patients.

### 3.4. Complications and Follow-Up

In patient 1, no complications were observed and the patient was discharged from the hospital six weeks after ALDLT. One year after ALDLT she had good liver function without signs for rejection or dysfunction of the graft. 

In the second patient, who suffered from decompensated liver cirrhosis stage Child C, a necrosis of the biliary duct evolved and relaparotomy was performed on the 15th postoperative day. He subsequently had severe biliary peritonitis with septic shock. Repeated infection and septic multiorgan failure including impaired liver function made long-term treatment in the critical care unit necessary, and the patient died on the 110th postoperative day. Repeated liver biopsies and the bile duct specimen showed no signs of antibody or complement deposit. 

Patient 4 had no in-hospital complications and was discharged on the 55th postoperative day. Subsequently, he had cholestasis due to anastomotic stricture and endoscopic stent placement was necessary. Cholestasis resolved and bilirubin returned to the normal range. He is alive with good liver function more than one year after ALDLT. 

In all three patients, AMR was excluded histologically by allograft biopsies taken at different time points and, in patient 2, at autopsy. Standard methods for light microscopy and immunohistochemistry did not reveal signs of antibody or cell mediated rejection, that is, deposition of immunglobulins or complement [19].

## 4. Discussion

Here we describe successful ABO-incompatible ALDLT in three patients applying different protocols, including pre- and posttransplant apheresis, reinforced immunosuppression, and/or splenectomy, without any occurrence of AMR while concurrently presenting the problems and challenges of the management of such patients. 

The largest cohort of patients undergoing ABO-incompatible ALDLT has been reported by Egawa and colleagues [12]. In 136 patients registered by the Japan Study Group for ABO-incompatible transplantation he could show a significant decrease of AMR and increase of survival between 2000 and 2006 owing to improved immunosuppressive treatment and complication management. 

Although we were aware of the increased risks associated with ABO incompatibility, we decided to provide ABO-incompatible ALDLT to patients with immediate need for an allograft, which was not adequately reflected by the standard allocation procedure. Especially patients suffering from malignant tumors of the liver may not receive a liver graft within the standard allocation procedure because of their suboptimal prognosis compared to patients with cirrhosis. If the patient and the potential donor are informed about the prognosis and risks of ALDLT under the given circumstances, we evaluated all patients in need for a liver graft and transplantation was performed only because no other potential ABO-compatible living donor or deceased donor was available. 

Under beneficial conditions ABO incompatible ALDLT has been conducted without special treatment, but this is associated with an increased risk of AMR [31]. We thus felt obliged to treat our first two patients according to a protocol that included repeated plasmapheresis, splenectomy, and reinforced immunosuppression [11]. The first patient we transplanted applying these strategies had unconstrained graft function and an uncomplicated course without any signs of rejection. Long-term monitoring of the isoagglutinin titers showed stable low titers after two preoperative apheresis sessions. 

Patient 2 underwent the same protocol but died three months after ALDLT. The necrosis of the biliary duct may possibly be due to technical failure but the patient suffered from decompensated alcoholic liver cirrhosis and was in critical condition before ALDLT. Bile duct necrosis due to ABO incompatibility is unlikely since no signs for AMR was seen in liver biopsies and in the bile duct specimen. But the subsequent clinical course may also be attributed to ABO-incompatible ALDLT. He suffered from recurrent and severe systemic infection, which finally led to septic multiorgan failure and death. It has been recognized that splenectomy and plasmapheresis in combination with reinforced immunosuppression may lead to overimmunosuppression and may make patients prone to severe systemic infection [11, 17]. 

In light of this, we decided to change our protocol to minimize morbidity related to immunosuppression. Recently, rituximab, a monoclonal humanized antibody against the target CD20, has been applied for ABO-incompatible kidney [24, 25, 32, 33] and liver transplantation [21, 27, 34] in lieu of splenectomy. Patient 3 was thus treated with rituximab after extrahepatic spread of malignant disease had been excluded. As an additional immunosuppression, we utilized mycophenolic acid instead of sirolimus because it may be more effective in inhibiting expansion of the B cell pool [35, 36]. Despite repeated plasmapheresis and immunoadsorbtion, isoagglutinin titers could not be reduced below the target level for ALDLT, because after each session the patient experienced a rapid rebound of isoagglutinin titers. This phenomenon has also been described before [24, 37], but the exact mechanism remains to be elucidated. Therefore, we finally decided against transplantation in the face of donor risk and increased risk of graft failure. Unfortunately, no blood group compatible graft could be allocated to this patient within the next three months and the patient suffered from extrahepatic spread of malignant disease. 

In our last patient, we used ATG for the induction of immunosuppression. ATG interferes with T-cell dependent activation of alloreactive B-cells by removing CD4+ T-cell help; and it has been shown to trigger apoptosis not only in T-cells but also in B-cells [29] and may thus be more effective than rituximab. Hence, ATG has been used effectively to treat AMR [38] and for induction in ABO-incompatible kidney transplantation [30]. In our last patient, we applied a treatment regimen of 1.5 mg per kg body weight starting at ALDLT and continued until postoperative day ten, a protocol that has been evaluated for living donor kidney transplantation by Gloor et al. [30]. In this patient, preoperative isoagglutinin titers were lowered successful, but after ALDLT titers increased steadily without clinical signs of rejection or mitigation of liver function. Effective reduction of titers was only sustained by repeated plasmapheresis. After four plasmapheresis sessions, we decided against further treatment because under a favorable donor-recipient blood group combination, that is, mismatches for donor blood group A2 or B [39–41], high pre- and postoperative titer levels may be tolerated without increasing AMR or graft loss [15, 16]. Subsequently no AMR or alteration of graft function was seen in this patient, although the relevant titer (A2) remained elevated for almost half a year before spontaneously declining below pretransplant level (see Figure 2(b)). A similar spontaneous decline or stable reduction below pretransplant levels could also be observed in the other two patients; this has also been reported by other authors, suggesting graft accommodation or even tolerance [17, 42]. Deletion and/or anergy have been proposed as possible mechanisms, but adsorbtion of antibodies by graft antigen may also be possible. 

Optimal treatment of patients after ALDLT should include triple immunosuppression (i.e., tacrolimus, mycophenolat mofetil, and prednisolone), pre- and postoperative plasmapheresis or immunoadsorbtion targeting isoagglutinin titers of 1 : 16 or lower, and induction with rituximab or ATG. We do not think that splenectomy and portal vein or hepatic artery infusion is necessary.

## 5. Conclusion

We have successfully performed three ABO-incompatible ALDLT with different protocols. Protocols were changed because the three ALDLTs were performed over a period of six years and there have been many changes in the immunosuppressive treatment after ABO-incompatible ALDLT. At first sight this heterogeneity may limit generalizability of our findings but also may provide new insight into the possibilities and limitations of these different protocols. Despite differences in treatment all patients had good initial graft function and no signs of rejection after ALDLT and two of the three patients had a long-term patient and graft survival. Indeed, further improvement is warranted and the different strategies should be evaluated in multicenter studies to assess their efficacy and safety. Nonetheless, ABO incompatible ALDLT should be offered to all patients in cases of immediate need for an allograft without the possibility to allocate a blood group compatible organ.

## Figures and Tables

**Table 1:** Clinical characteristics of recipients. HCC: hepatocellular carcinoma; HCV: hepatitis C virus.

Case	Sex	Age at ALDLT	Diagnosis and indication for ALDLT	Child-Pugh status	ABO donor-recipient match
1	F	61	bile duct carcinoma	A	A2 → O
2	M	44	alcoholic cirrhosis	C	B → O
3	M	51	cholangiocellular carcinoma	A	A2B → O
4	M	48	HCC in HCV cirrhosis	B	A2B → B
